# Molecular simulations of MOF membranes for separation of ethane/ethene and ethane/methane mixtures†
†Electronic supplementary information (ESI) available: List of the MOFs studied in this work and their structural properties. Potential parameters of gas molecules used in the simulations. See DOI: 10.1039/c7ra11562h


**DOI:** 10.1039/c7ra11562h

**Published:** 2017-11-10

**Authors:** Cigdem Altintas, Seda Keskin

**Affiliations:** a Department of Chemical and Biological Engineering, Koc University, Rumelifeneri Yolu, Sariyer, 34450, Istanbul, Turkey. Email: skeskin@ku.edu.tr

## Abstract

Metal organic framework (MOF) membranes have been widely investigated for gas separation applications. Several MOFs have been recently examined for selective separation of C_2_H_6_. Considering the large number of available MOFs, it is not possible to fabricate and test the C_2_H_6_ separation performance of every single MOF membrane using purely experimental methods. In this study, we used molecular simulations to assess the membrane-based C_2_H_6_/C_2_H_4_ and C_2_H_6_/CH_4_ separation performances of 175 different MOF structures. This is the largest number of MOF membranes studied to date for C_2_H_6_ separation. We computed adsorption selectivity, diffusion selectivity, membrane selectivity and gas permeability of MOFs for C_2_H_6_/C_2_H_4_ and C_2_H_6_/CH_4_ mixtures. Our results show that a significant number of MOF membranes are C_2_H_6_ selective for C_2_H_6_/C_2_H_4_ separation in contrast to traditional nanoporous materials. Selectivity and permeability of MOF membranes were compared with other membrane materials, such as polymers, zeolites, and carbon molecular sieves. Several MOFs were identified to exceed the upper bound established for polymeric membranes and many MOF membranes exhibited higher gas permeabilities than zeolites and carbon molecular sieves. Examining the structure–performance relations of MOF membranes revealed that MOFs with cavity diameters between 6 and 9 Å, porosities lower than 0.50, and surface areas between 500–1000 m^2^ g^–1^ have high C_2_H_6_ selectivities. The results of this study will be useful to guide the experiments to the most promising MOF membranes for efficient separation of C_2_H_6_ and to accelerate the development of new MOFs with high C_2_H_6_ selectivities.

## Abstract 2

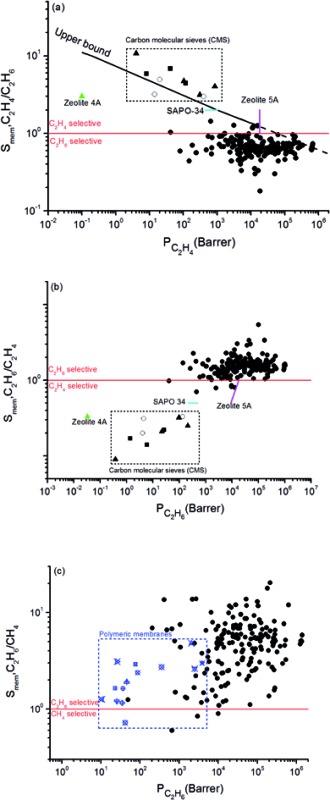
Molecular simulations were used to assess the membrane-based C_2_H_6_/C_2_H_4_ and C_2_H_6_/CH_4_ separation performances of 175 different MOF structures.

## 1. Introduction

The separation of different members of the C_2_ hydrocarbon family has industrial importance because these molecules are primary feedstocks for various chemicals.^1^ Ethane/ethene (C_2_H_6_/C_2_H_4_) separation is generally carried out using distillation. This is one of the most energy intensive single distillations practiced commercially. Separation of C_2_H_6_ from methane (CH_4_) similarly requires energy intensive distillation operations. The energy and equipment costs associated with these gas separations could be significantly reduced by the development of alternative separation methods.^2^ Adsorption-based and membrane-based gas separations provide very large reductions in the energy consumption and costs of these processes. The greatest limitation in the applications of adsorption and membrane-based gas separation technologies is the low selectivity of the materials used as adsorbents and membranes. Research on identification of new materials that can achieve C_2_H_6_ separations with high selectivity has gained significant attention in the last decade.

Metal organic frameworks (MOFs) are considered as a new class of nanoporous materials that can be used as adsorbents and membranes in various gas separations. MOFs are composed of metal ions connected with organic ligands.^3^ They have well-defined pore structures,^4^ large surface areas (500–6500 m^2^ g^–1^),^5^ high porosities, good thermal and mechanical stabilities which make them strong candidates for gas separation applications.^6^,^7^ MOFs have been widely studied for adsorption of CO_2_, CH_4_, and H_2_ in addition to the separation of several gas mixtures including CO_2_/CH_4_, CH_4_/H_2_, CO_2_/N_2_, CO_2_/H_2_ and noble gases.^8^–^12^ Most of the studies related to the gas separation with MOFs in the literature focused on the CO_2_ capture whereas research on C_2_H_6_ separation using MOFs has recently started. Several experimental studies measured single-component adsorption isotherms of CH_4_, C_2_H_4_, and C_2_H_6_ in various MOFs and these initial studies showed that MOFs can be promising materials for C_2_H_6_ separations.^13^–^23^

Considering the large number of available MOFs, it is not practical to identify the most promising adsorbent materials using purely experimental manners. Most of the works used molecular simulations to study MOFs for adsorption-based C_2_H_6_ separations. Guo *et al.* used molecular simulations to study C_2_H_6_/CH_4_ separation in IRMOF-1 (isoreticular metal organic framework) and four different zeolitic imidazolate frameworks, ZIFs (ZIF-8, ZIF-71, ZIF-80, ZIF-90).^24^ They showed that ZIFs exhibit better C_2_H_6_/CH_4_ separation performance compared to MOR zeolite and IRMOF-1. Wu *et al.* studied four different ZIFs using molecular simulations and reported their C_2_H_6_/C_2_H_4_ selectivities.^25^ Pillai *et al.* carried out molecular simulations to explore C_2_H_6_/C_2_H_4_ separation in interpenetrated and non-interpenetrated IRMOF-8 and reported C_2_H_6_ selectivities.^26^ We recently performed the first large-scale molecular simulation study for C_2_H_6_/C_2_H_4_ and C_2_H_6_/CH_4_ separations using MOFs and reported adsorption selectivity of 278 different MOFs.^27^ Our results showed that there is a large number of MOFs that exhibit significantly higher adsorption selectivity than zeolites for separation of C_2_H_6_/C_2_H_4_ and C_2_H_6_/CH_4_ mixtures. All these studies suggest that MOFs have strong potential to be used in adsorption-based C_2_H_6_/C_2_H_4_ and C_2_H_6_/CH_4_ separations.

Membrane-based C_2_H_6_ separation is an alternative to the adsorption-based separation. Conventional polymeric membranes cannot achieve the desired performance, combination of high selectivity and high permeability, required for C_2_H_6_ separation.^28^–^31^ Polymers with high selectivity exhibit low permeability and polymers that have high gas permeability suffer from low selectivity. Due to this trade-off, recent studies have been directed towards developing more advanced membrane materials for C_2_H_6_ separations. Identification of MOF membranes that can achieve both high selectivity and high permeability will be very useful to replace polymeric membranes with MOFs. However, we have very limited information about the membrane-based C_2_H_6_ separation potential of MOFs. The number of studies focusing on C_2_H_6_/C_2_H_4_ and C_2_H_6_/CH_4_ separations with MOF membranes is scarce in the literature. Only two different types of MOFs were used as membranes for C_2_H_6_/C_2_H_4_ separation. Bux *et al.* predicted membrane selectivity of ZIF-8 as the product of adsorption and diffusion selectivities for an equimolar C_2_H_6_/C_2_H_4_ mixture.^15^ They reported that C_2_H_6_ adsorbs stronger than C_2_H_4_ but C_2_H_4_ diffuses faster and overcompensates the adsorption preference for C_2_H_6_, resulting in a MOF membrane that is weakly selective for C_2_H_4_. Pan and Lai reported single-component permeances of CH_4_, C_2_H_4_ and C_2_H_6_ through ZIF-8 membranes.^32^ Caro's group reported single-component permeances of CH_4_ and C_2_H_6_ for ZIF-90 membranes.^33^ MOFs were recently used as filler particles in polymers to fabricate mixed matrix membranes (MMMs) in order to improve C_2_H_6_ and C_2_H_4_ permeabilities of polymers.^34^–^37^

Predicting separation performances of MOF membranes requires diffusion coefficients of C_2_H_6_/C_2_H_4_ and C_2_H_6_/CH_4_ mixtures through the pores of MOFs. Stallmach *et al.* reported intra-crystalline self-diffusion of CH_4_ and C_2_H_6_ in MOF-5 (also known as IRMOF-1) using pulsed field gradient (PFG) NMR technique.^38^ Ford *et al.* reported self-diffusivity of CH_4_ and C_2_H_6_ in MOF-5 using experiments and molecular simulations.^39^ Chmelik *et al.* studied diffusion of C_2_H_6_/C_2_H_4_ mixtures in ZIF-8 using different NMR techniques and showed that C_2_H_4_ diffusion is 5 times faster than C_2_H_6_ diffusion.^40^ Molecular simulations were used to compute self-diffusivity of CH_4_ in MOF-5.^9^,^41^,^42^ Borah *et al.* recently conducted molecular dynamics simulations to predict diffusion behavior of pure CH_4_ and C_2_H_6_ in 6 different MOFs.^43^ Self-diffusivities of C_2_H_6_,^44^ C_2_H_4_,^45^ and transport diffusivities of CH_4_, C_2_H_4_, and C_2_H_6_ (ref. 46) in ZIF-8 were computed by molecular dynamics simulations. All these simulations were generally carried out for single-component gases since calculating diffusivities of gas mixtures is computationally demanding. We recently reported diffusion coefficients of C_2_H_6_/C_2_H_4_ and C_2_H_6_/CH_4_ binary mixtures in 5 different MOFs and using this diffusion data we predicted membrane selectivities and gas permeabilities of the 5 MOFs for C_2_H_6_ separations.^27^ Our results on a small number of MOFs demonstrated that MOFs are promising membranes for preferential separation of C_2_H_6_ from C_2_H_4_ due to their higher selectivities and higher gas permeabilities compared to zeolites and polymers.

The literature we summarized above shows that current studies on MOF membranes for C_2_H_6_ separations examine only a few types of structures. There is currently no large-scale computational study to identify the separation performances of different MOFs for C_2_H_6_/C_2_H_4_ and C_2_H_6_/CH_4_ mixtures. Considering the large variety and number of available MOFs, there may be many existing MOFs with better separation performances, high membrane selectivities and high gas permeabilities. In order to identify membrane-based C_2_H_6_ separation performances of a large number of MOFs, we used molecular simulations and computed adsorption equilibria and self-diffusivities of C_2_H_6_/C_2_H_4_ and C_2_H_6_/CH_4_ mixtures in 175 different MOFs. Using this data, we predicted adsorption selectivity, diffusion selectivity, membrane selectivity and gas permeability of 175 MOFs both for C_2_H_6_/C_2_H_4_ and C_2_H_6_/CH_4_ separations. Results were compared with well-known membrane materials, polymers, zeolites and carbon molecular sieves to evaluate the potential of MOFs. Relations between adsorption selectivity, diffusion selectivity and membrane selectivity of MOFs were discussed to understand the individual effects of adsorption and diffusion on the membranes' performances. We finally examined the relations between easily computable structural properties such as pore size, surface area and porosity of MOFs and their selectivities to provide structure–performance relationships that can serve as a map for experimental synthesis of new MOFs with better gas separation performances.

## 2. Computational details

### 2.1. MOFs

We used the same MOF database that we considered in our previous work.^27^ This database was originally prepared using the solvent-free MOF database constructed by Chung and coworkers^47^ and adding some well-known MOFs taken from our previous studies^48^ in order to cover widely studied subfamilies such as ZIFs, covalent organic frameworks (COFs), and bio-MOFs. This database does not have any MOF with open metal sites (OMS) in order to eliminate the necessity of performing detailed quantum-level calculations to accurately define the specific interactions between C_2_H_4_ and OMS of MOFs as discussed in detail before.^27^,^49^ We then refined our database to only include MOFs that have pore sizes (largest cavity diameters) larger than the kinetic diameters of the C_2_H_6_, C_2_H_4_ and CH_4_ molecules so that all these gases can enter into the MOFs' pores and diffuse. After this elimination, we ended up with 175 different MOF structures. All crystal structures of MOFs were taken from the Cambridge Crystallographic Data Centre (CCDC).^50^ The complete list of MOFs including the CCDC names and common names is given in Table S1 of ESI.† Structural properties of materials such as pore limiting diameter (PLD), largest cavity diameter (LCD), pore volume, porosity, and surface area were computed using Poreblazer algorithm^51^ in which the Dreiding force field^52^ was utilized. In this algorithm, He and N_2_ atoms were used as probe molecules for pore size and surface area calculations, respectively. The sigma parameters for He and N_2_ were used in their default values in the Poreblazer algorithm as 2.58 Å and 3.31 Å, respectively. The cut-off distance and cubelet size were used as 12.8 Å and 0.2 Å, respectively. The largest anticipated pore diameter was increased to 20 Å and the size of the bin was decreased to 0.25 Å in that algorithm.

### 2.2. Molecular simulations

Grand Canonical Monte Carlo (GCMC)^53^ simulations were used to compute binary adsorption isotherms of C_2_H_6_/C_2_H_4_ and C_2_H_6_/CH_4_ mixtures in MOFs. In a GCMC simulation, adsorbed amounts of each gas component were calculated by specifying the bulk pressure, temperature and composition of the bulk gas mixture. The Dreiding force field^52^ was used for the MOFs. In cases where the potential parameters of atoms were not available from the Dreiding force field, these parameters were taken from the Universal Force Field (UFF).^54^ These force fields were selected based on the results of our initial simulation studies that give a good agreement with the available experimental uptake data of C_2_H_6_, C_2_H_4_ and CH_4_ in various MOFs as reported in our previous work.^27^ Single-site spherical Lennard-Jones (LJ) 12–6 potential was used to model CH_4_ (ref. 55) whereas two-site spherical LJ potentials were used for C_2_H_6_ and C_2_H_4_ molecules following the literature (given Table S2†).^25^ C_2_H_6_ and C_2_H_4_ molecules were described as uncharged united-atom models with one pseudo-atom representing –CH_3_ group and –CH_2_ group that was located at the position of carbon atom similar to the TraPPE united atom force field.^55^ Since the adsorbate molecules did not contain any dipole, the long-range electrostatic contribution was omitted following the previous studies in the literature.^25^ The cut-off distance for truncation of the intermolecular interactions was set to 12 Å for GCMC simulations. A simulation box of 2 × 2 × 2 crystallographic unit cells was used. Periodic boundary conditions were applied in all simulations. During the simulations, 1.5 × 10^7^ steps were performed to guarantee the equilibration and 1.5 × 10^7^ steps were performed to sample the desired properties.

Computing membrane properties of MOFs requires diffusivities of gas molecules in the pores of materials. In order to obtain self-diffusivities of C_2_H_6_/C_2_H_4_ and C_2_H_6_/CH_4_ mixtures in MOFs, we performed Equilibrium Molecular Dynamics (EMD) simulations. The initial states of EMD simulations with the appropriate gas loadings were obtained from the GCMC simulations and each system was equilibrated for 20 ps prior to taking data. The Nosé–Hoover thermostat was applied to run EMD simulations at NVT (constant number of molecules, volume and temperature) ensemble.^53^ At least 10 independent EMD simulations with a length of 16 ns were performed to compute self-diffusivities of gases at the given loadings. The estimated uncertainties of the self-diffusivities were at least one order of magnitude smaller than the reported diffusion coefficients. More details of using GCMC and EMD simulations to obtain adsorption data and diffusion coefficients in various MOFs can be found in our previous studies.^56^,^57^

Molecular simulations should be performed for multiple materials on time scales shorter than the same materials can be assessed experimentally. Since we considered a large number of MOF membranes in this work, we used rigid framework assumption. Almost all molecular simulations for MOF membranes in the literature used this assumption because it saves a significant amount of computational time. Recent studies showed that including lattice flexibility does not make any significant change in the gas adsorption results of MOFs that have pore sizes larger than the guest molecules.^58^–^60^ Chmelik *et al.* could not find any evidence for gate-opening effect or another structural transitions of ZIF-8 upon adsorption of C_2_H_6_/C_2_H_4_ mixture.^40^ On the other hand, lattice flexibility can be important for the diffusion of large gas molecules in the MOFs having narrow windows.^44^,^46^ We recently carried out flexible EMD simulations to examine the effect of MOF's flexibility on the predicted membrane performance.^61^ Considering flexibility of the framework made a negligible effect on the gas permeability and selectivity of the MOFs having large pores whereas more pronounced changes were seen in gas permeabilities of the materials having narrow pores. Another recent study on Xe separations showed that flexibility should be considered in shape selective screening studies for the highest degree of accuracy and to achieve the best ranking of high-performance materials.^62^ Since MOFs considered in this work were specifically chosen to have larger pore diameters than the kinetic diameters of the three gas molecules we studied, flexibility effects were expected to be small and they were not taken into account for computational efficiency. The idea of our calculations is that once the potential value of a membrane material has been demonstrated by molecular simulations, further detailed studies such as flexible simulations can be performed to increase the precision of initial assessment.

### 2.3. Calculation of membrane properties

Adsorption selectivities (*S*_ads_) of MOFs for C_2_H_6_/C_2_H_4_ and C_2_H_6_/CH_4_ separations were calculated using the results of mixture GCMC simulations as we previously reported.^27^ *S*_ads_ is defined as the ratio of compositions of the adsorbed gases (*x*) in the adsorbent material normalized by the ratio of bulk phase compositions (*y*) of component *i* to component *j*:

1 *S*_ads(*i*/*j*)_ = (*x*_*i*_/*x*_*j*_)/(*y*_*i*_/*y*_*j*_)

Adsorption selectivities of MOFs were computed at 10 bar, 298 K for equimolar C_2_H_6_/C_2_H_4_ and C_2_H_6_/CH_4_ mixtures. The ratio of self-diffusivities of gases obtained from the EMD simulations was used to define the diffusion selectivity of MOFs. Diffusion selectivity, (*S*_diff_) was calculated as the ratio of the self-diffusivities (*D*_*i*,self_) of each gas component in the binary mixture where *c*_*i*_ represents the corresponding adsorbed loading of gas species *i* calculated from the GCMC simulations at 10 bar, 298 K:2
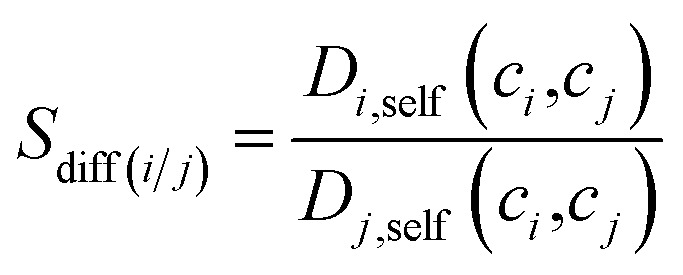



Once the adsorption and diffusion selectivities of MOFs were computed for a given gas mixture, membrane selectivity (*S*_mem_), also known as permeation selectivity, was calculated as the multiplication of adsorption selectivity and diffusion selectivity at a membrane feed pressure of 10 bar as described in the literature.^56^ The validity of this model was shown by comparing its predictions with the experimentally measured selectivity and permeability data of MOF membranes for various gas separations in previous studies.^56^

3 *S*_mem(*i*/*j*)_ = *S*_ads(*i*/*j*)_ × *S*_diff(*i*/*j*)_

Not only high selectivity but also high gas permeability is required for an efficient and economic membrane process. Therefore, we also computed gas permeabilities through MOFs using the following expression,^63^4
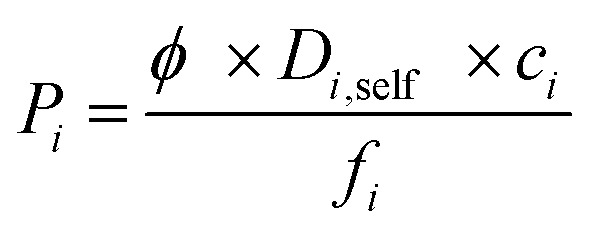
where *P*_*i*_, *φ*, *D*_*i*,self_, *c*_*i*_ and *f*_*i*_ represent the permeability of the component *i* (mol m^–1^ s^–1^ Pa^–1^), the porosity of MOFs (given in Table S1†), the self-diffusivity of the component *i* in the mixture (m^2^ s^–1^), the concentration of component *i* at the upstream face of the membrane (mol m^–3^) and the bulk phase fugacity of the component *i* (Pa), respectively. The bulk gas compositions of C_2_H_6_/C_2_H_4_ and C_2_H_6_/CH_4_ mixtures were assumed to be equimolar in all molecular simulations because Guo *et al.* recently showed that composition does not strongly affect the C_2_H_6_ selectivity of a MOF material.^24^ The accuracy of our computational approach to predict the membrane performances of MOFs for various gas mixtures using the GCMC and EMD data as explained above was shown in several of our previous studies by comparing the results of simulations with the experiments.^64^,^65^ We recently reported remarkably well agreement between our simulations and experimental measurements both for single-component and mixture CH_4_ permeability through different MOF membranes including MOF-5, ZIF-78, ZIF-95.^61^

## 3. Results and discussion

### 3.1. Membrane properties of MOFs

We validated the accuracy of our GCMC simulations to predict the adsorption of C_2_H_6_, C_2_H_4_ and CH_4_ in various MOFs such as CuBTC, PCN-16, Co-MOF-74 and Mg-MOF-74 by comparing results of our molecular simulations with the available experimental data of different research groups in our previous work.^27^ In this work, we aimed to validate the accuracy of our EMD simulations for the diffusivity of C_2_H_6_ and CH_4_ in the MOFs' pores. There is limited information about the diffusivity of these gases in MOFs due to the difficulty of measurement of self-diffusivity using purely experimental techniques and high computational demands of molecular simulations. Table 1 compares C_2_H_6_ and CH_4_ diffusivities calculated from our molecular simulations with the available experimental and computational data taken from the literature. Our simulated data for C_2_H_6_ and CH_4_ diffusivities in MOFs agreed well with the previous simulation studies of different research groups. C_2_H_6_ diffusivities in MOF-5 predicted by our molecular simulations agreed well with the experimental measurements of Stallmach *et al.*^38^ and Ford *et al.*^39^ whereas simulations predicted an order of magnitude slower CH_4_ diffusivity in MOF-5 compared to the experiments. This discrepancy was attributed to the imperfections in the micropore structure which influenced the experimental studies but which were not taken into account in the EMD simulations.^38^,^39^ Overall, the good agreement between our simulations and reported values in the literature for diffusion of C_2_H_6_ and CH_4_ in MOFs suggests that simulated diffusion coefficients can be used to model gas transport through the MOF membranes.

**Table 1:** Comparison of simulated self-diffusivities of gases in MOFs with the literature

MOF		This work		Other simulations		Ref.	Experiments		Ref.
		*D*_CH_4__ (cm^2^ s^–1^)	*D*_C_2_H_6__ (cm^2^ s^–1^)	*D*_CH_4__ (cm^2^ s^–1^)	*D*_C_2_H_6__ (cm^2^ s^–1^)		*D*_CH_4__ (cm^2^ s^–1^)	*D*_C_2_H_6__ (cm^2^ s^–1^)	
NU-125	Single	3.89 × 10^–4^	7.87 × 10^–5^	2–3 × 10^–4^	1–1.7 × 10^–4^	43			
	Binary	3.39 × 10^–4^	1.47 × 10^–4^	1.2 × 10^–4^	1.2 × 10^–4^	43			
PCN-14	Single	2.10 × 10^–4^	7.62 × 10^–5^	1–1.5 × 10^–4^	0.3–1 × 10^–4^	43			
	Binary	1.92 × 10^–4^	8.02 × 10^–5^	1 × 10^–4^	6 × 10^–5^	43			
COF-10	Single	9.88 × 10^–4^		7.7 × 10^–4^		66			
MOF-5	Single	3.37 × 10^–4^	1.51 × 10^–4^	3 × 10^–4^	1.5 × 10^–4^	39	2 × 10^–3^	1.8 × 10^–4^	39
	Single			3.1 × 10^–4^		41	1.7 × 10^–3^	2.1 × 10^–4^	38
	Single			3.08 × 10^–4^		42			
	Single			3.5 × 10^–4^		9			

**Fig. 1:**
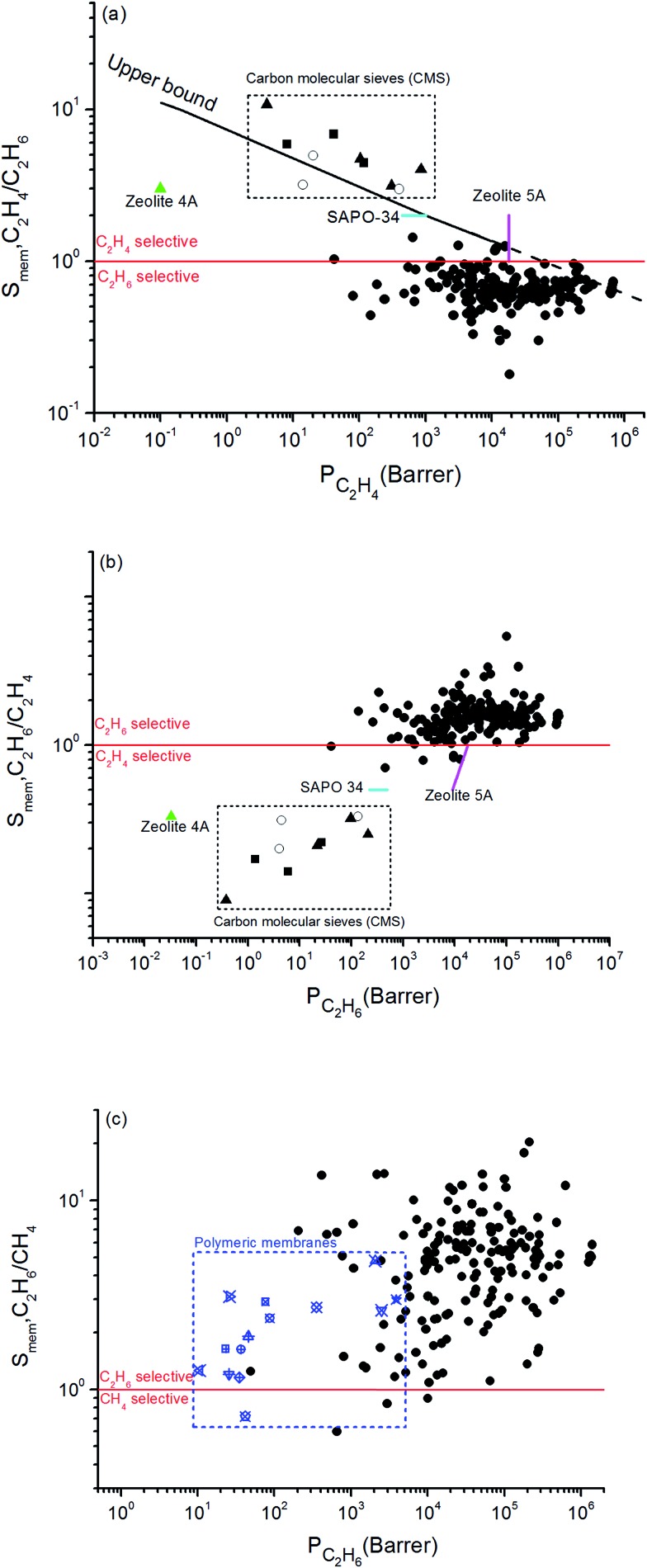
Selectivity and permeability of MOFs for (a) C_2_H_4_/C_2_H_6_, (b) C_2_H_6_/C_2_H_4_, (c) C_2_H_6_/CH_4_ separations. Data for CMSs shown within the box^69^–^71^ and data for zeolites shown with color symbols^75^–^77^ were taken from the literature in (a) and (b). Data for polymeric membranes shown with blue symbols in (c) were taken from the literature.^34^,^73^,^74^,^78^–^81^

Combining adsorption data obtained from the GCMC simulations and diffusion data obtained from the EMD simulations, we computed selectivity and permeability of MOF membranes for C_2_H_6_/C_2_H_4_ and C_2_H_6_/CH_4_ mixture separations as shown in Fig. 1. In order to compare MOFs with traditional membrane materials, we collected selectivity and permeability data of zeolites, carbon molecular sieves (CMSs) and polymers for C_2_H_6_/C_2_H_4_ separations. At that point it is important to reiterate that membrane materials that preferentially select C_2_H_6_ over C_2_H_4_ are very scarce. Zeolites, CMSs and polymers are generally C_2_H_4_ selective. In order to be consistent with the literature data, we showed C_2_H_4_/C_2_H_6_ selectivity and C_2_H_4_ permeability of MOF membranes in Fig. 1(a). Traditional polymeric membranes, such as Matrimid, 4,4′-(hexafluoroisopropylidene)dipthalicanhydride-2,4,6-trimethyl-1,3-phenylene diamine (6FDA-DAM), 4,4′-(hexafluoroisopropylidene)dipthalicanhydride:3,3′,4,4′-biphenyltetracarboxylic dianhydride-2,4,6-trimethyl-1,3-phenylene diamine (6FDA:BPDA-DAM) selectively separate C_2_H_4_ from C_2_H_6_, generally due their sorption selectivities.^67^ The black solid line in Fig. 1(a) represents the experimental C_2_H_4_/C_2_H_6_ upper bound for polymers which was established by Rungta *et al.*^67^ based on the pure gas permeability data, similar to the Robeson's upper bound.^68^ Polymeric membranes are mostly located below this bound and it is highly desired to identify new membrane materials that can exceed this bound by exhibiting higher selectivity and/or higher permeability than polymers. Since MOFs are highly porous materials compared to polymers, C_2_H_4_ permeabilities of MOF membranes are significantly higher than those of polymers. According to the upper bound, the C_2_H_4_ permeabilities of polymeric membranes are in the range of 0.1–10^4^ Barrer whereas MOF membranes we considered in this work exhibit C_2_H_4_ permeabilities in the range of 42–6.75 × 10^5^ Barrer. Therefore, we extrapolated the Robeson's upper bound with a dashed line in Fig. 1(a) to show the high C_2_H_4_ permeabilities of MOFs. In fact, 4 MOFs, YUTYOC, IDIWOH, OWITAQ and OWITUQ were found to exceed the upper bound due to their high C_2_H_4_ permeabilities.

The red solid line shows the selectivity preference of the membranes in Fig. 1(a). MOFs located above (below) this line are C_2_H_4_ (C_2_H_6_) selective. A significant number of MOF membranes was identified to show C_2_H_6_ selectivity over C_2_H_4_ and these MOFs were located below the red line. Well-known zeolites such as zeolite 4A, zeolite 5A, SAPO-34 and CMSs are C_2_H_4_ selective membranes in C_2_H_6_/C_2_H_4_ separations.^67^ For example, zeolite 4A membrane has C_2_H_4_ selectivity of 3 and C_2_H_4_ permeability of 0.1 Barrer whereas the MOFs we considered in this work are mostly C_2_H_6_ selective with significantly higher C_2_H_4_ permeabilities.^67^ The maximum C_2_H_4_ selectivity of CMS membranes was reported to be around 10 and their maximum C_2_H_4_ permeabilities were around 1000 Barrer.^69^–^71^ A recent study reported that a ZIF-8-filled 6FDA-DAM MMM exhibit C_2_H_4_ selectivity of 3.2 and permeability of 72.9 Barrer depending on the ZIF loading in the polymer.^72^ All these comparisons show that C_2_H_4_ permeability of MOF membranes are significantly higher than that of zeolite 4A, zeolite 5A, SAPO-34, and ZIF-8-filled MMM. Since majority of the MOFs we examined in this work are C_2_H_6_ selective, we additionally showed the C_2_H_6_ selectivity and C_2_H_6_ permeability of MOFs in Fig. 1(b). This figure shows that MOF membranes can selectively separate C_2_H_6_ from C_2_H_4_ with high selectivity. 169 out of 175 MOFs are C_2_H_6_ selective with selectivities in the range of 1.0–5.4. Among these MOFs, EYOPUE has the highest C_2_H_6_/C_2_H_4_ selectivity (5.4) and OWITIY has the highest C_2_H_6_ permeability (1.04 × 10^6^ Barrer). Selectivity of 11 MOFs was found to be slightly larger than unity which means they do not have a strong preference for C_2_H_6_ or C_2_H_4_ and therefore they cannot be used as selective membranes for C_2_H_6_/C_2_H_4_ separations. A small number of MOF membranes (6 out of 175) was identified to show low/mediocre C_2_H_4_ selectivity over C_2_H_6_ and located below the red line.

Fig. 1(c) represents C_2_H_6_/CH_4_ selectivity and C_2_H_6_ permeability of MOF membranes. Although an upper bound is not established yet, several polymeric membranes were tested for C_2_H_6_/CH_4_ separation and we collected this data from the literature to compare MOF membranes with polymers.^34^,^71^–^76^ Most of the polymeric membranes exhibit C_2_H_6_ permeabilities between 10–100 Barrer and C_2_H_6_/CH_4_ selectivities between 0.7 and 3. Only polydimethylsiloxane (PDMS) and polympentanamer (PPM) membranes show higher C_2_H_6_ permeabilities reaching to 2070 Barrer (ref. 73) and 3900 Barrer,^74^ respectively. Most of the MOFs studied in this work exhibit higher C_2_H_6_ permeabilities and higher C_2_H_6_/CH_4_ selectivities than these polymers. Among 175 MOFs, all the MOFs except 3 of them (XENZUN, GITVAH and YARYEV) were identified to be C_2_H_6_ selective over CH_4_ and their C_2_H_6_ permeabilities were computed to be in the range of 49.5–1.39 × 10^6^ Barrer. The most selective MOF for C_2_H_6_/CH_4_ separation was identified as NEXXIZ, with a selectivity of 20.5 and C_2_H_6_ permeability of 2.12 × 10^5^ Barrer. OWITAQ was identified as the most permeable MOF with C_2_H_6_ permeability of 1.39 × 10^6^ Barrer and C_2_H_6_/CH_4_ selectivity of 6. All these results indicate that MOFs are highly promising membrane materials for preferential separation of C_2_H_6_ from CH_4_.

**Fig. 2:**
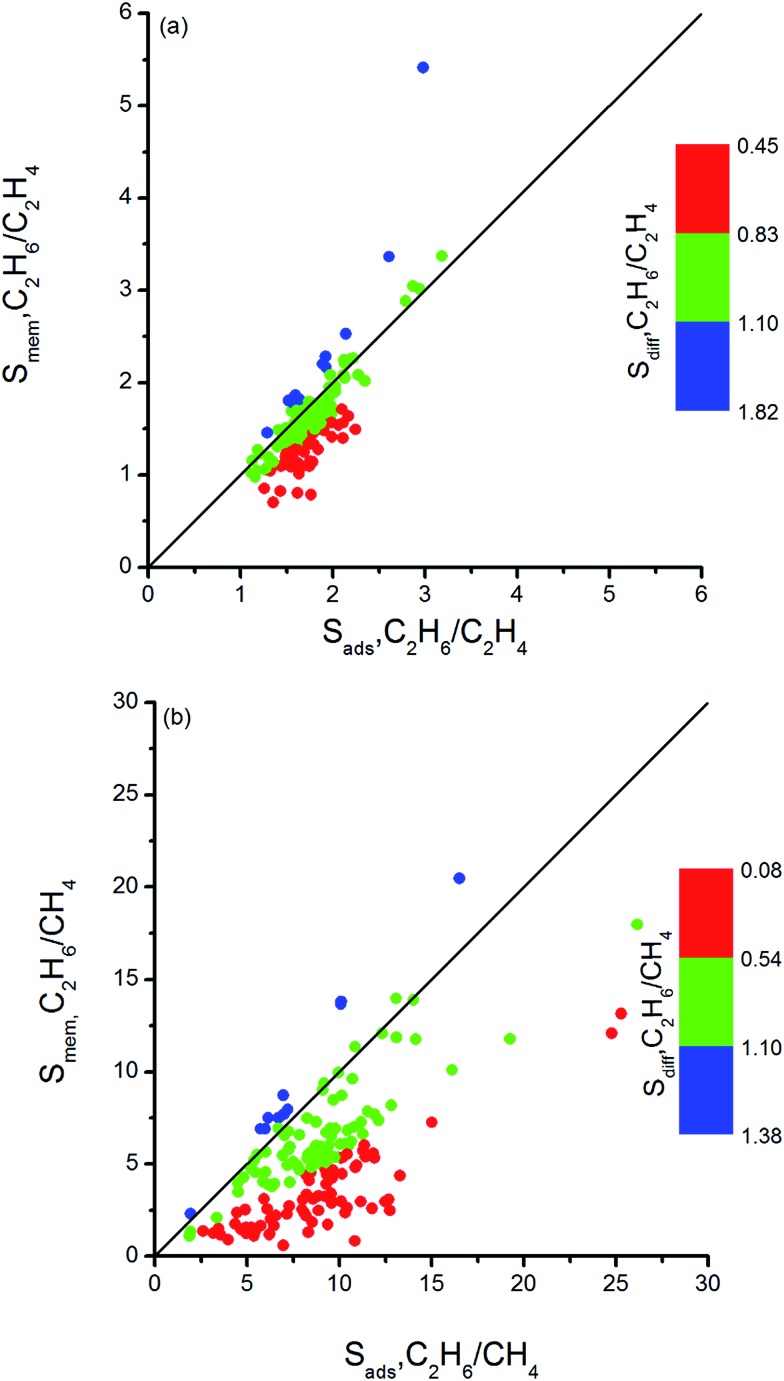
Adsorption, diffusion and membrane selectivities of MOFs for (a) C_2_H_6_/C_2_H_4_ and (b) C_2_H_6_/CH_4_ separations.

Combination of adsorption and diffusion selectivity determines the membrane selectivity of a MOF. In order to evaluate the individual effects of gas adsorption and diffusion on the membrane performance of MOFs, we examined the relation between adsorption, diffusion, and membrane selectivity in Fig. 2. All selectivities were computed for equimolar gas mixtures. The colored dots in Fig. 2(a) show the distribution of the diffusion selectivities of MOFs for C_2_H_6_/C_2_H_4_ mixture. The LJ energy parameter was higher for C_2_H_6_ (*ε*_C_2_H_6__/*k*_B_ = 108 K) than C_2_H_4_ (*ε*_C_2_H_4__/*k*_B_ = 92.8 K) to reflect stronger dispersion interactions. Since C_2_H_6_ is energetically preferred over C_2_H_4_, C_2_H_6_ (C_2_H_4_) is the strongly (weakly) adsorbed component in all MOFs. Therefore, adsorption selectivities favor C_2_H_6_ over C_2_H_4_ (C_2_H_6_/C_2_H_4_ selectivity > 1) for all MOFs. Diffusion selectivities favor C_2_H_4_ (C_2_H_6_/C_2_H_4_ selectivity < 1) in most of the MOFs since C_2_H_4_ molecules diffuse faster than C_2_H_6_ molecules. C_2_H_4_ molecules are lighter, smaller and weakly adsorbed into the pores of MOFs which leads to faster diffusion of C_2_H_4_ than C_2_H_6_. For 50 MOFs shown by red color, the diffusion selectivity for C_2_H_6_ over C_2_H_4_ is ranged from 0.45 to 0.83. Since the membrane selectivity was estimated as the multiplication of the adsorption and diffusion selectivities, the predicted membrane selectivities of these MOFs for C_2_H_6_ are lower than their adsorption selectivities as shown in Fig. 2(a). In other words, these MOFs are more useful in adsorption-based C_2_H_6_/C_2_H_4_ separations than in membrane-based separations in terms of C_2_H_6_ selectivity. 113 MOFs shown by green color have diffusion selectivities around unity (0.83–1.10), which means diffusion does not strongly favor one gas species over other in the mixture. Since diffusion selectivities are close to one, membrane selectivities of these MOFs are only slightly lower than their adsorption selectivities. On the other hand, 12 of the MOFs shown by blue color exhibit diffusion selectivities higher than unity (1.11–1.82). That means self-diffusion coefficient of C_2_H_6_ is slightly higher than C_2_H_4_. In this case, both adsorption and diffusion favors the same component, C_2_H_6_ over C_2_H_4_ in the mixture. As a result, membrane selectivities of these MOFs are higher than their adsorption-based selectivities. In fact, it is highly desired to find materials in which both adsorption and diffusion favor the same gas component and lead to high membrane selectivities. Table 2 summarizes the top ten C_2_H_6_ selective MOF membranes together with their adsorption, diffusion, membrane selectivities in addition to the self-diffusivities of each gas species in these materials. Both adsorption and diffusion selectivities of the MOFs listed in Table 2 are higher than 1, which means C_2_H_6_ is favored over C_2_H_4_ by both mechanisms. For example, EYOPUE has both high adsorption selectivity and high diffusion selectivity for C_2_H_6_ over C_2_H_4_. As a result it was identified as the most selective MOF membrane. Therefore, it is more useful to utilize the MOFs listed in Table 2 as membranes rather than as adsorbents for selective separation of C_2_H_6_ from C_2_H_4_.

**Table 2:** Top ten MOF membranes for C_2_H_6_/C_2_H_4_ separation

MOF	*S*_ads,_ C_2_H_6_/C_2_H_4_	*D*_C_2_H_6__ (cm^2^ s^–1^)	*D*_C_2_H_4__ (cm^2^ s^–1^)	*S*_diff_, C_2_H_6_/C_2_H_4_	*S*_mem_, C_2_H_6_/C_2_H_4_	*P*_C_2_H_6__ (Barrer)	*P*_C_2_H_4__ (Barrer)
EYOPUE	2.98	1.45 × 10^–4^	7.97 × 10^–5^	1.82	5.41	1.01 × 10^5^	1.86 × 10^4^
EYOPOY	3.18	1.62 × 10^–4^	1.53 × 10^–4^	1.06	3.37	1.73 × 10^5^	5.12 × 10^4^
AMAFUQ	2.61	8.09 × 10^–5^	6.27 × 10^–5^	1.29	3.36	4.42 × 10^4^	1.32 × 10^4^
YUVSUE	2.87	1.56 × 10^–5^	1.47 × 10^–5^	1.06	3.04	1.60 × 10^4^	5.25 × 10^3^
CAYDOX	2.94	1.28 × 10^–4^	1.25 × 10^–4^	1.02	3.01	4.95 × 10^4^	1.64 × 10^4^
CAYGIU	2.79	9.65 × 10^–5^	9.34 × 10^–5^	1.03	2.88	3.71 × 10^4^	1.29 × 10^4^
BUSNAF	2.14	2.84 × 10^–5^	2.41 × 10^–5^	1.18	2.53	1.24 × 10^4^	4.92 × 10^3^
TUSGUJ	1.92	3.80 × 10^–6^	3.19 × 10^–6^	1.19	2.28	6.05 × 10^3^	2.65 × 10^3^
UHAXUW	2.22	6.58 × 10^–7^	6.45 × 10^–7^	1.02	2.26	3.40 × 10^2^	1.50 × 10^2^
NEXXIZ	2.18	1.27 × 10^–4^	1.23 × 10^–4^	1.03	2.25	1.45 × 10^5^	6.44 × 10^4^

It is interesting to discuss the MOFs for which the diffusion selectivity for C_2_H_4_ overcompensates the adsorption selectivity for C_2_H_6_ and makes the membrane C_2_H_4_ selective. We listed adsorption, diffusion, membrane selectivities and gas permeabilities of the C_2_H_4_ selective MOFs in Table 3. All these five MOFs are weakly selective for C_2_H_4_. For example, XENZUN was predicted to show the highest membrane selectivity. Adsorption weakly favors C_2_H_6_ over C_2_H_4_ in this MOF whereas diffusion favors C_2_H_4_ over C_2_H_6_ and dominates the adsorption selectivity. AVEROJ has a low C_2_H_4_/C_2_H_6_ adsorption selectivity but the diffusion selectivity strongly favors C_2_H_4_ over C_2_H_6_ and makes the membrane C_2_H_4_ selective. KEXFAU has the highest adsorption selectivity for C_2_H_4_/C_2_H_6_ separation as can be seen from Table 3, but its diffusion selectivity is close to unity making the membrane weakly selective for C_2_H_4_. These examples signify the importance of diffusion selectivity in governing membrane's separation performance. If the adsorption selectivity does not strongly favor one component in the mixture, then diffusion selectivity determines the membrane's gas separation performance.

**Table 3:** Top MOF membranes for C_2_H_4_/C_2_H_6_ separation

MOF	*S*_ads,_ C_2_H_4_/C_2_H_6_	*D*_C_2_H_4__ (cm^2^ s^–1^)	*D*_C_2_H_6__ (cm^2^ s^–1^)	*S*_diff_, C_2_H_4_/C_2_H_6_	*S*_mem_, C_2_H_4_/C_2_H_6_	*P*_C_2_H_4__ (Barrer)	*P*_C_2_H_6__ (Barrer)
XENZUN	0.74	7.62 × 10^–7^	3.94 × 10^–7^	1.93	1.43	6.47 × 10^2^	4.53 × 10^2^
AVEROJ	0.57	5.47 × 10^–6^	2.44 × 10^–6^	2.24	1.27	3.12 × 10^3^	2.45 × 10^3^
TUDJOS	0.62	2.22 × 10^–5^	1.10 × 10^–5^	2.01	1.25	1.59 × 10^4^	1.28 × 10^4^
YARYEV	0.70	2.19 × 10^–5^	1.26 × 10^–5^	1.74	1.21	1.17 × 10^4^	9.65 × 10^3^
KEXFAU	0.80	2.15 × 10^–5^	1.46 × 10^–5^	1.47	1.17	1.12 × 10^4^	9.60 × 10^3^

Similar selectivity analysis was done for C_2_H_6_/CH_4_ separation in Fig. 2(b). Adsorption strongly favors C_2_H_6_ and strongly adsorbed C_2_H_6_ molecules move slower than weakly adsorbed, lighter CH_4_ molecules. As a result, diffusion selectivity favors CH_4_ over C_2_H_6_ and becomes less than 1 for almost all MOFs as shown by red and green points in Fig. 2(b). Since adsorption strongly favors C_2_H_6_ and diffusion weakly favors CH_4_, membrane-based C_2_H_6_ selectivities of MOFs are less than their adsorption-based selectivities. There are 11 MOFs shown by blue color in Fig. 2(b) in which diffusivity of C_2_H_6_ is slightly higher than the diffusivity of CH_4_. Our EMD simulations showed that gas molecules generally diffuse only in one direction in these MOFs and the high number of slowly diffusing C_2_H_6_ molecules hinders the fast diffusion of CH_4_ molecules in the pores. As a result, diffusion selectivities are around 1.1–1.3 and these MOFs are promising membrane materials since both adsorption and diffusion favors the same component C_2_H_6_ over CH_4_. Performances of the top ten promising MOF membranes for selective separation of C_2_H_6_ from CH_4_ were summarized in Table 4. For example, adsorption and diffusion favor C_2_H_6_ over CH_4_ in NEXXIZ, TIRQOB, ZUQPOQ, UHAXUW whereas high adsorption selectivity towards C_2_H_6_ dominates the diffusion selectivity towards CH_4_ in other MOFs as shown in Table 4.

**Table 4:** Top ten MOF membranes for C_2_H_6_/CH_4_ separation

MOF	*S*_ads,_ C_2_H_6_/CH_4_	*D*_C_2_H_6__ (cm^2^ s^–1^)	*D*_CH_4__ (cm^2^ s^–1^)	*S*_diff_, C_2_H_6_/CH_4_	*S*_mem_, C_2_H_6_/CH_4_	*P*_C_2_H_6__ (Barrer)	*P*_CH_4__ (Barrer)
NEXXIZ	16.49	1.41 × 10^–4^	1.14 × 10^–4^	1.24	20.46	2.12 × 10^5^	1.04 × 10^4^
EYOPOY	26.16	1.36 × 10^–4^	1.98 × 10^–4^	0.69	17.97	1.81 × 10^5^	1.01 × 10^4^
TIRQOB	13.07	1.81 × 10^–6^	1.70 × 10^–6^	1.07	13.98	2.72 × 10^3^	1.95 × 10^2^
CAYDOX	14.01	1.10 × 10^–4^	1.11 × 10^–4^	0.99	13.88	5.20 × 10^4^	3.74 × 10^3^
ZUQPOQ	10.10	3.71 × 10^–6^	2.72 × 10^–6^	1.37	13.81	2.18 × 10^3^	1.58 × 10^2^
UHAXUW	10.08	6.34 × 10^–7^	4.68 × 10^–7^	1.35	13.65	4.15 × 10^2^	3.04 × 10^1^
EYOPUE	25.29	1.14 × 10^–4^	2.20 × 10^–4^	0.52	13.10	1.01 × 10^5^	7.68 × 10^3^
LUMDIG	24.77	2.01 × 10^–5^	4.12 × 10^–5^	0.49	12.08	2.81 × 10^4^	2.33 × 10^3^
SUTBIT	12.32	2.13 × 10^–4^	2.17 × 10^–4^	0.98	12.07	6.31 × 10^5^	5.23 × 10^4^
CAYGIU	13.09	1.12 × 10^–4^	1.23 × 10^–4^	0.91	11.86	5.31 × 10^4^	4.48 × 10^3^

**Fig. 3:**
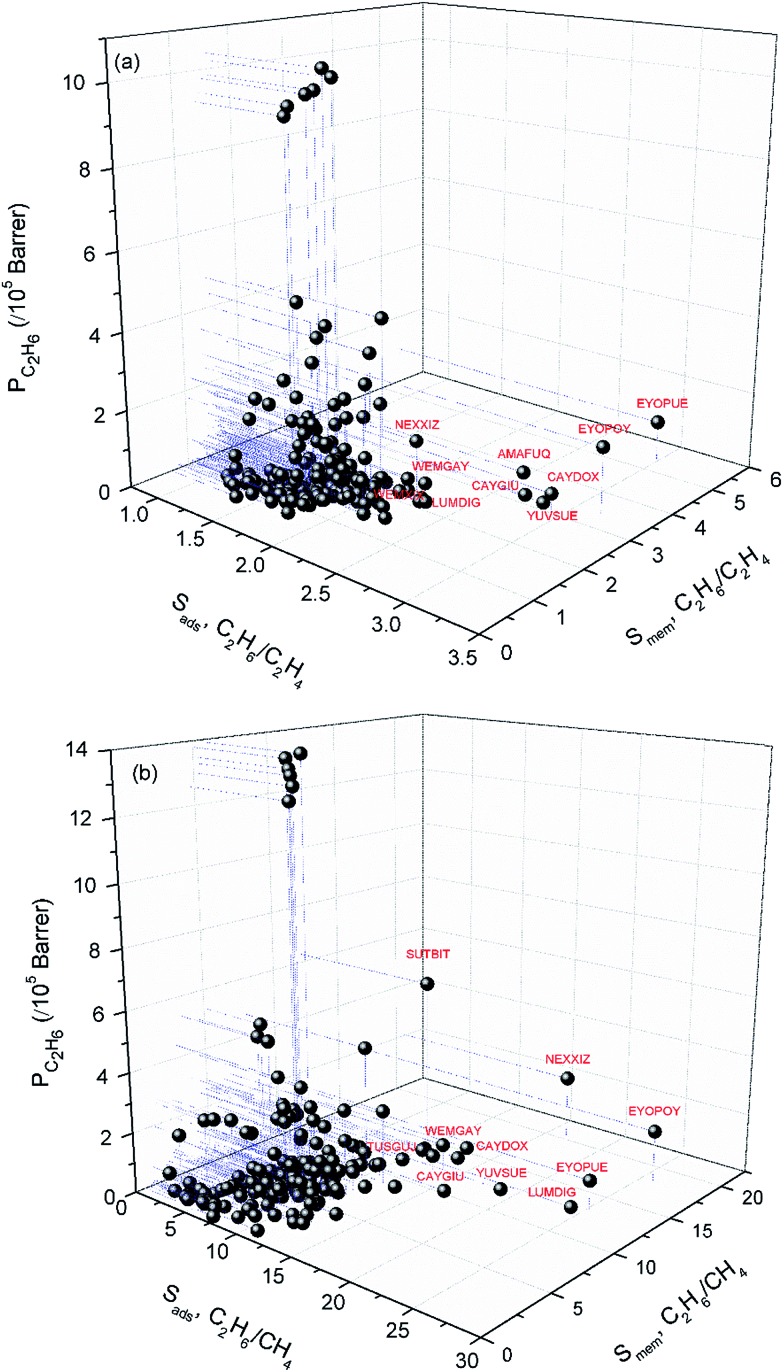
Adsorption selectivity, membrane selectivity and C_2_H_6_ permeability of MOFs for (a) C_2_H_6_/C_2_H_4_ and (b) C_2_H_6_/CH_4_ separations. Top ten MOFs for (a) C_2_H_6_/C_2_H_4_ and (b) C_2_H_6_/CH_4_ separations are labelled with red labels. Blue dashed lines are to guide the eye.

As we discussed above, some MOFs are promising for adsorption-based gas separations whereas some others are good candidates for membrane-based gas separations. We aimed to identify the MOFs that can be used both as effective adsorbents and membranes for the preferential separation of C_2_H_6_ from C_2_H_4_ and CH_4_. Selectivity is generally considered as the most critical factor to assess equilibrium and kinetic-based separation performances of materials. High adsorption selectivity is desired for adsorbents whereas both high selectivity and permeability are required for membranes. Therefore, we considered these three performance metrics, adsorption selectivity, membrane selectivity and permeability of the desired gas in order to identify the most promising MOFs that can be used both as adsorbents and membranes. For C_2_H_6_/C_2_H_4_ separation, adsorption and membrane selectivities of MOFs were computed to be 1.1–3.2 and 0.7–5.4, respectively and C_2_H_6_ permeabilities of MOFs were predicted to be 41–1.04 × 10^6^ Barrer. For C_2_H_6_/CH_4_ separation, adsorption and membrane selectivities were calculated to be 1.9–26.2 and 0.60–20.5, respectively with C_2_H_6_ permeabilities of 49.5–1.39 × 10^6^ Barrer. We set the minimum adsorption and membrane selectivity to 2 and showed the top ten MOFs with the highest C_2_H_6_ permeabilities for C_2_H_6_/C_2_H_4_ separation in Fig. 3(a). Similarly, for C_2_H_6_/CH_4_ separation, we considered the MOFs that have adsorption and membrane selectivities larger than 10. After these two constraints we identified the MOFs with the highest C_2_H_6_ permeabilities in Fig. 3(b). Results show that MOFs named as EYOPOY, NEXXIZ, EYOPUE, CAYDOX, WEMGAY, CAYGIU, LUMDIG and YUVSUE are common in the top ten promising material list of C_2_H_6_/C_2_H_4_ and C_2_H_6_/CH_4_ separations. For example, EYOPOY has high adsorption-based selectivity both for C_2_H_6_/C_2_H_4_ (3.2) and C_2_H_6_/CH_4_ (26.2) in addition to high membrane-based selectivity both for C_2_H_6_/C_2_H_4_ (3.4) and C_2_H_6_/CH_4_ (18). This result suggests that these 8 MOFs can be used as effective adsorbents and membranes for the selective separation of C_2_H_6_ from C_2_H_4_ and CH_4_.

### 3.2. Structure–performance relations for MOFs

**Fig. 4:**
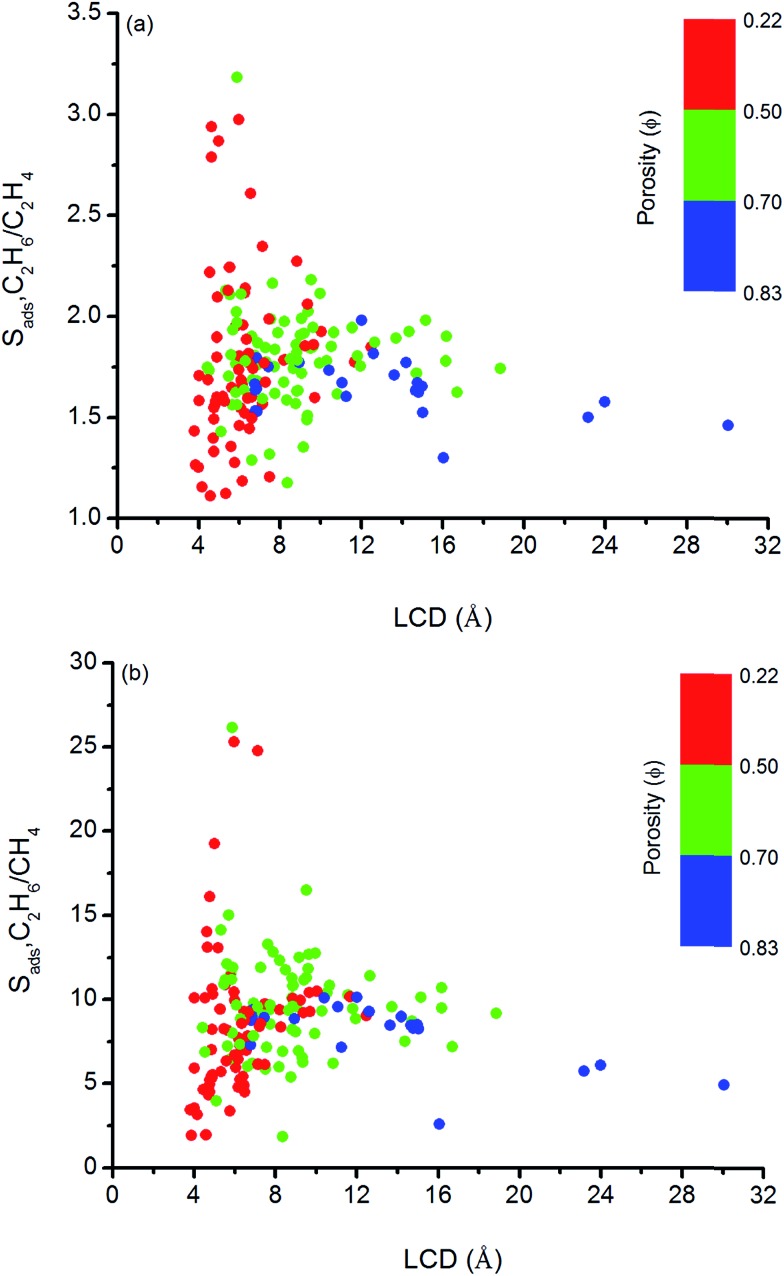
Relation between adsorption selectivities and LCDs of MOFs as a function of porosities for (a) C_2_H_6_/C_2_H_4_ and (b) C_2_H_6_/CH_4_ separations.

We so far focused on the gas separation performance of MOFs as adsorbents and as membranes. Establishing relation between structures and separation performances of MOFs would be very useful to save computational time and to guide the experimental studies for the synthesis of materials with the desired topology. However, clear identification of this type of relations is challenging because separation performance of a material is determined by the interplay of various factors such as chemical topology, porosity, pore size and shape and it cannot be simply correlated to only a single or two structural properties.^82^ In order to simply structure–performance analysis, we examined the relation between selectivity and easily computable structural properties of MOFs such as pore size, porosity, and surface area. Fig. 4 shows that there is a correlation between adsorption selectivity and LCD as well as porosity of MOFs. MOFs with LCDs around 4.5–6 Å generally exhibit higher C_2_H_6_/C_2_H_4_ and C_2_H_6_/CH_4_ selectivities (>2 and >10, respectively) than MOFs with larger pore sizes. As the LCD increases, selectivity generally decreases. MOFs that have large LCDs (>6 Å) have lower C_2_H_6_/C_2_H_4_ and C_2_H_6_/CH_4_ selectivities (<2 and <10, respectively) since both gas molecules can easily pass through the pores. Fig. 4 also shows that increasing porosity decreases the adsorption selectivity and this is the common outcome for both gas separations. The porosity of MOFs we considered in this work ranges from 0.22 to 0.83. Although the color labeling is not distinct in Fig. 4, it is clear that MOFs with porosity lower than 0.50 exhibit higher selectivity.

**Fig. 5:**
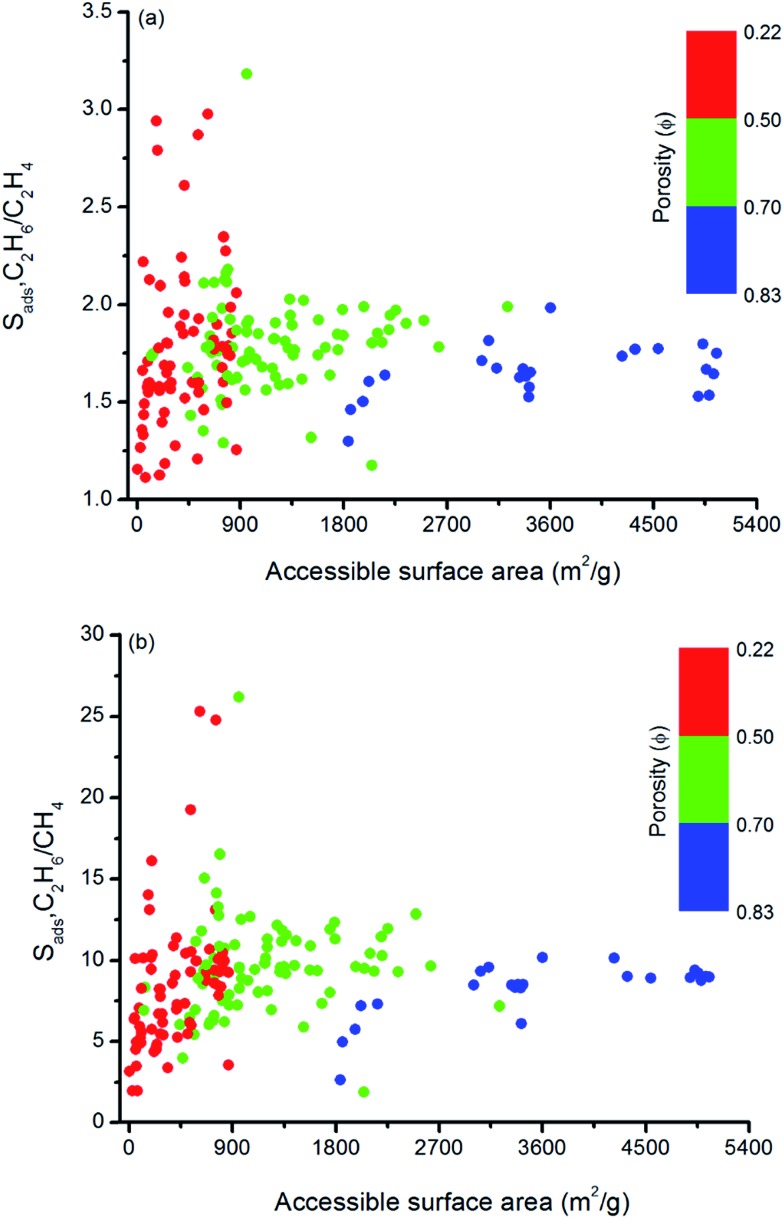
Relation between adsorption selectivities and surface areas of MOFs as a function of porosities for (a) C_2_H_6_/C_2_H_4_ and (b) C_2_H_6_/CH_4_ separations.

Similarly, Fig. 5 shows that MOFs having lower surface areas exhibit higher adsorption selectivity. For example, MOFs with surface areas in the range of 500–1000 m^2^ g^–1^ and 750–1000 m^2^ g^–1^ tend to show adsorption selectivities higher than 2 and 10 for C_2_H_6_/C_2_H_4_ and C_2_H_6_/CH_4_ separations, respectively. Overall, results of our structure–performance analysis suggest that MOFs with LCDs around 4.5–6 Å, porosities less than 0.50, and surface areas in the range of 500–1000 m^2^ g^–1^ can be potentially promising adsorbents for efficient C_2_H_6_ separations.

**Fig. 6:**
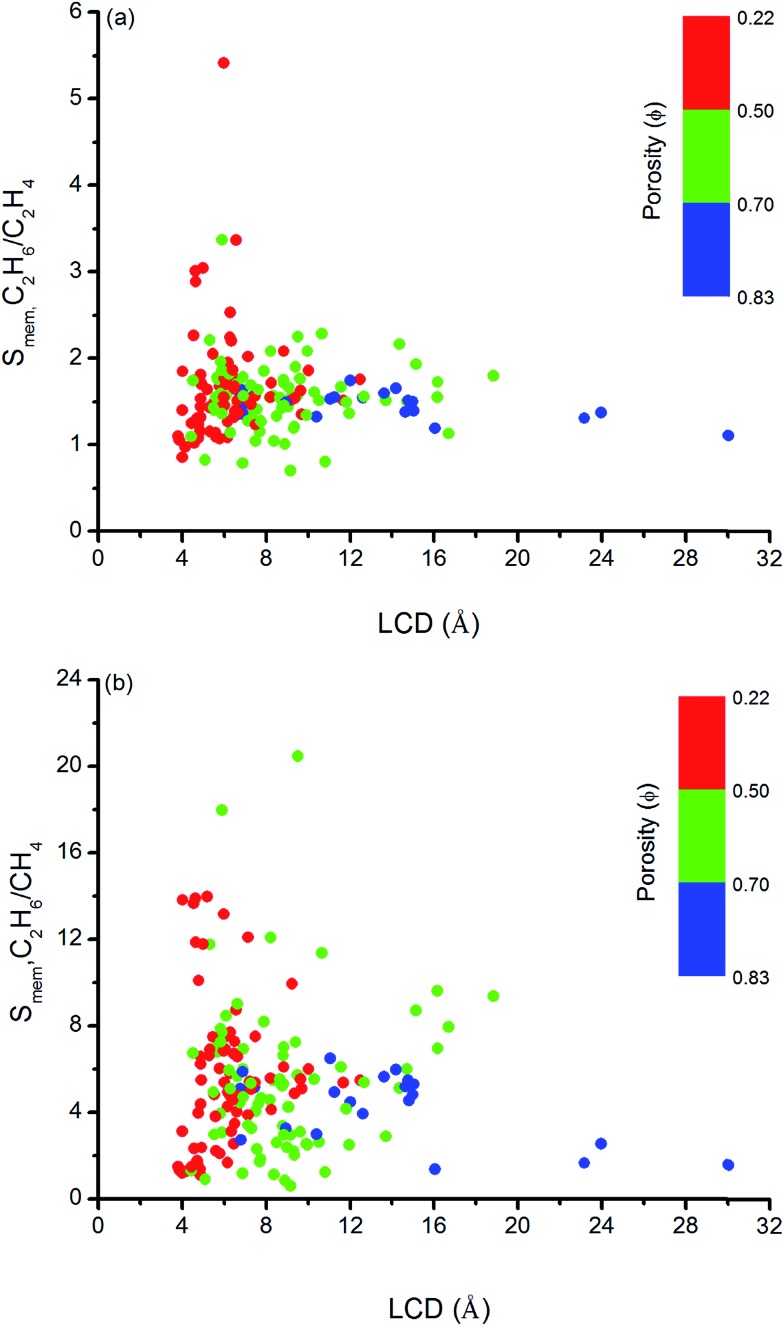
Relation between membrane selectivities and LCDs of MOFs as a function of porosities for (a) C_2_H_6_/C_2_H_4_ and (b) C_2_H_6_/CH_4_ separations.

**Fig. 7:**
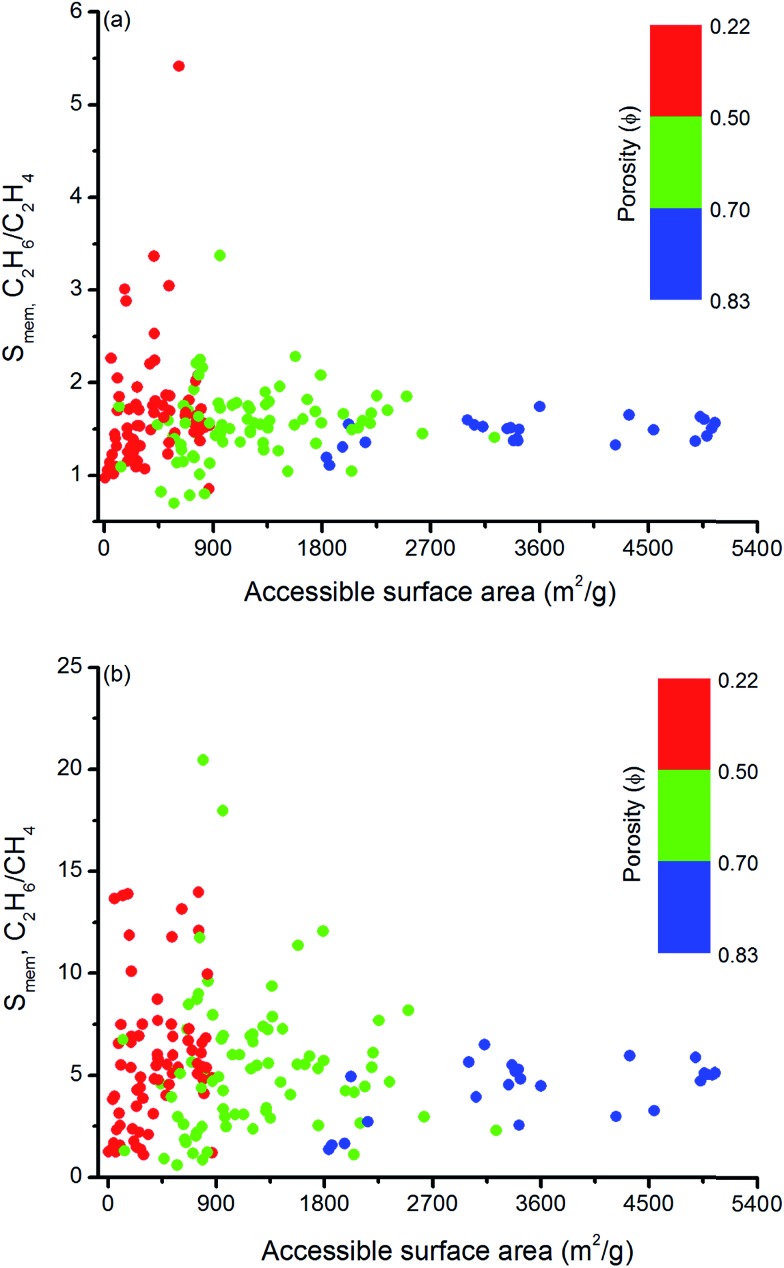
Relation between membrane selectivities and accessible surface areas of MOFs as a function of porosities for (a) C_2_H_6_/C_2_H_4_ and (b) C_2_H_6_/CH_4_ separation.

Similar structure–performance analysis was carried out to unlock the relation between membrane selectivity of MOFs, pore sizes, porosities and surface areas. Fig. 6 shows that membranes with LCDs in the range of 6–7 Å and 6–9 Å are more selective for separation of C_2_H_6_ from C_2_H_4_ and CH_4_, respectively. It is also observed that among the two MOFs with close LCDs but different porosities, the MOF with lower porosity generally have higher membrane selectivity. For example, EYOPUE and SUBDOI have close LCDs (5.97 Å and 6.29 Å) but different porosities (0.46 and 0.56). The one with the lower porosity exhibits high membrane selectivity (5.41) whereas the other one has low membrane selectivity (1.14). This example underlines the importance of the material's porosity on the membrane selectivity. Similar to the adsorption selectivity, as the surface area and porosity decrease, membrane selectivity increases as shown in Fig. 7. For C_2_H_6_/C_2_H_4_ and C_2_H_6_/CH_4_ separation, we obtained the highest membrane selectivities (5.4 and 20.5, respectively) when the surface areas of the MOFs are between 500–1000 m^2^ g^–1^ and 750–1000 m^2^ g^–1^, respectively.

**Fig. 8:**
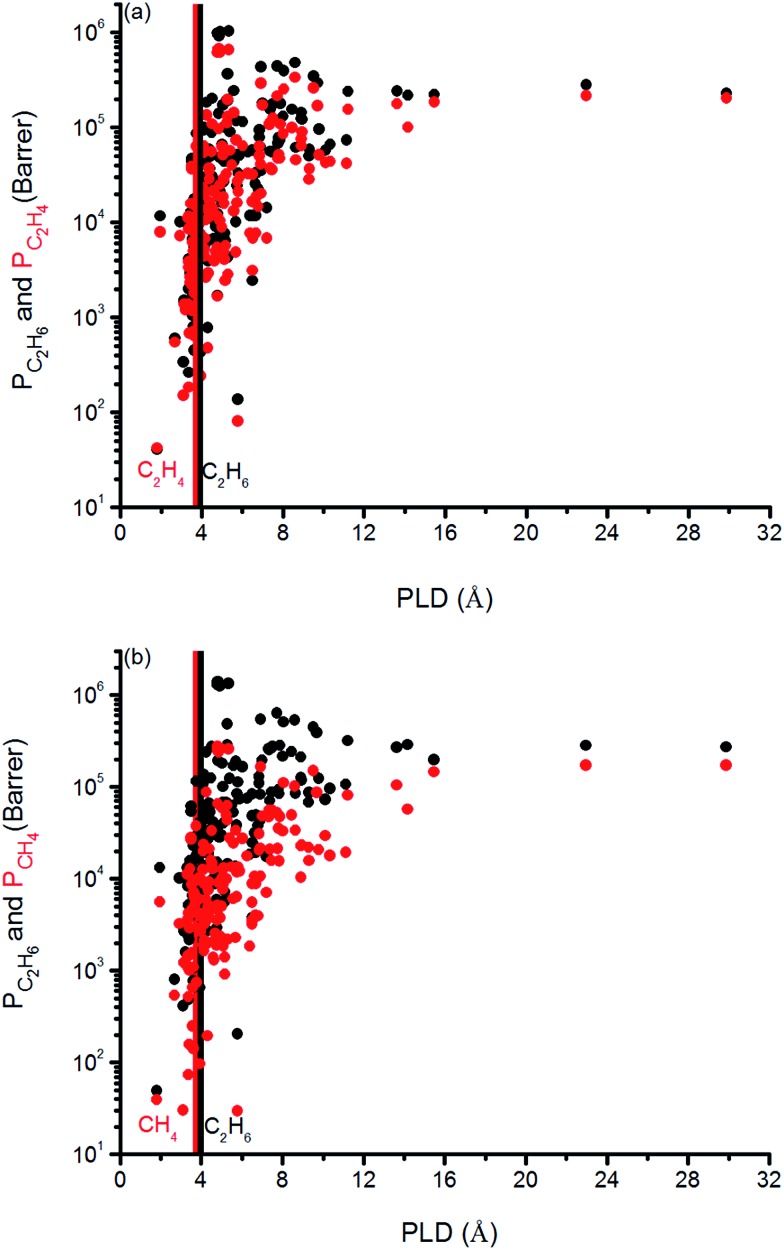
Relation between gas permeabilities and PLDs of MOFs for (a) C_2_H_6_/C_2_H_4_ and (b) C_2_H_6_/CH_4_ separations. Black symbols represent the C_2_H_6_ permeabilities, red symbols represent permeabilities of C_2_H_4_ in (a) and CH_4_ in (b).

We finally investigated the effect of pore size on the gas permeabilities of MOFs in Fig. 8. Vertical solid lines in this figure represent the kinetic diameter of the gas molecules present in the mixture. We specifically focused on the PLD, the smallest pore diameter in the structure, rather than LCD since we refined our MOF database to have the LCDs greater than the kinetic diameters of the gas molecules as we discussed before. It can be seen that once the PLD is slightly larger than the kinetic diameter of a gas molecule, permeability of that gas increases. In Fig. 8(a), C_2_H_6_ (C_2_H_4_) permeability increases from 10^5^ to 10^6^ (10^4^ to 10^5^) Barrer when PLD is larger than 3.76 (3.68) Å, which is the kinetic diameter of C_2_H_6_ (C_2_H_4_) molecule. In Fig. 8(b), C_2_H_6_ and CH_4_ permeabilities increase from 10^5^ to 10^6^ Barrer and from 10^4^ to 10^5^ Barrer for the MOFs that have PLD values slightly larger than 3.76 Å and 3.73 Å, the kinetic diameters of C_2_H_6_ and CH_4_, respectively. These increases can be attributed to the easier diffusion of gas molecules in the larger pores of MOFs. These results suggest that it is reasonable to choose MOFs with PLD values slightly larger than the kinetic diameters of the gas molecules that are desired to be separated in order to obtain high gas permeabilities.

## 4. Conclusions

In this study, we used GCMC and EMD simulations to compute adsorption and diffusion data of C_2_H_6_/C_2_H_4_ and C_2_H_6_/CH_4_ mixtures in 175 different MOFs. Using this data, membrane performances of MOFs were assessed for these two important gas separations. Majority of the MOFs we considered was identified as C_2_H_6_ selective membranes and a small number of MOFs was identified as C_2_H_4_ selective. This result is important since membranes that are selective for C_2_H_6_ over C_2_H_4_ are scarce in the literature and almost all traditional membrane materials such as polymers, zeolites and CMSs are C_2_H_4_ selective. MOF membranes that we considered in this work were found to exhibit higher gas permeabilities than these well-known membrane materials due to their highly porous structures. Examining the structure–performance relations of MOF membranes revealed that MOFs with porosities lower than 0.50, LCD values between 6–9 Å, and surface areas between 500–1000 m^2^ g^–1^ have the highest selectivities for C_2_H_6_/C_2_H_4_ and C_2_H_6_/CH_4_ separations.

The idea of our work was to identify promising MOF membranes for C_2_H_6_ separations using molecular simulations in order to direct experimental efforts, time and resources to those promising materials for experimental fabrication and testing of membranes under real operating conditions. It is very important to discuss the assumptions made in computational studies in order to evaluate the potential of new membrane materials in real applications. We assumed perfect MOF crystals in our GCMC and EMD simulations and predicted gas separation performances of MOFs as defect-free membranes. In reality, defects may be formed during membrane fabrication and they may reduce the membrane's expected selectivity. The idea of our calculations is that once the potential value of a membrane material has been demonstrated by molecular simulations, further experimental studies can be used to increase the precision of initial assessment. Our molecular simulations do not provide any information about the stability of MOFs, which is very important for real applications. An efficient membrane material must keep its structural stability under industrial operation conditions. This issue is more likely to be addressed experimentally. We searched for the stability information of the two MOFs, EYOPUE and NEXXIZ, which were identified as the top promising membrane material for selective separation of C_2_H_6_ from C_2_H_4_ and CH_4_, respectively. Experiments reported that they can retain their crystalline integrity at ambient conditions.^83^,^84^ The value of our computational work is that it can provide a motivation to perform detailed experimental studies for the thermal and structural stability of promising membrane materials. We believe that results of this work will guide experimental studies for the design and synthesis of new MOFs with better separation performances for C_2_H_6_ separations.

## Conflicts of interest

There are no conflicts to declare.

## Supplementary Material

Supplementary information
