# Supplementary material for: Molecular simulations of MOF membranes for separation of ethane/ethene and ethane/methane mixtures
Source: RSC Adv. 2017 Nov 10;7(82):52283–95. doi: 10.1039/c7ra11562h (PMC5735352; doi:10.1039/c7ra11562h)
Supplement: Supplementary file 1 [file RA-007-C7RA11562H-s001.pdf]

Electronic Supplementary Material (ESI) for RSC Advances.
This journal is © The Royal Society of Chemistry 2017

Supporting Information for

Molecular Simulations of MOF Membranes for Separation of Ethane/Ethene and Ethane/Methane Mixtures

Cigdem Altintas and Seda Keskin*

Department of Chemical and Biological Engineering, Koc University,

Rumelifeneri Yolu, Sariyer, 34450, Istanbul, Turkey

Submitted to *RSC Advances*

Table S1. List of the MOFs studied in this work and their structural properties.

#	MOF	Common name	PLD (Å)	LCD (Å)	Porosity	Density (g/cm^3)	Accessible surface area (m^2/g)
1	ACENIF		3.56	4.89	0.45	1.33	269.85
2	ACODED		3.38	5.52	0.49	1.13	385.45
3	AFUJUI		3.14	5.61	0.46	1.16	259.95
4	ALICEE		8.05	8.18	0.52	2.48	440.34
5	ALICII		8.61	8.77	0.55	1.98	568.55
6	ALOLOD	CPM-3	4.57	7.31	0.57	0.93	1314.94
7	AMAFUQ		5.57	6.57	0.39	1.55	411.44
8	AVEROJ		6.50	6.88	0.57	1.70	705.67
9	NUDLAA	BIOMOF-1	9.29	9.63	0.56	1.02	1056.46
10	BEYSEF	BIOMOF-12	3.96	4.89	0.48	1.18	698.78
11	BIYTEJ		5.77	6.06	0.48	1.39	540.21
12	BONMAT		4.49	5.82	0.59	0.87	1368.74
13	BUSMUY		5.16	6.61	0.43	1.26	484.20
14	BUSNAF		4.79	6.30	0.37	1.36	413.88
15	BUVWOF02		4.70	6.27	0.40	1.26	420.76
16	BUVXOG01		4.70	6.27	0.40	1.33	417.30
17	BUVYIB01		7.79	10.53	0.67	0.70	2097.83
18	CAYBAH		3.34	4.58	0.43	2.11	72.55
19	CAYDOX		4.40	4.63	0.37	1.49	167.61
20	CAYGIU		4.42	4.64	0.37	1.49	178.60
21	CECYOY		3.34	3.85	0.38	1.67	26.48
22	YUTYOC	COF-10	29.87	30.04	0.72	0.60	1861.31
23	YUTYIW	COF-12	7.84	8.34	0.51	1.00	1240.22
24	RAYQAK	COF-5	22.95	23.17	0.72	0.58	1968.71
25	CUGVUW		4.15	6.16	0.37	1.36	241.28
26	DIDBID		5.24	8.83	0.54	0.93	1189.28
27	DIDBOJ		5.29	8.82	0.54	0.92	1207.20
28	EBAMOL		3.61	7.71	0.56	1.05	649.94
29	ECAHIB		3.62	6.17	0.38	1.41	271.07
30	EJOGOZ		6.92	8.25	0.49	1.09	798.49
31	EMIHAK		3.96	5.79	0.49	1.69	412.01
32	EMIHIS	SCIF-9	5.76	9.40	0.67	1.48	1330.25
33	EXOTUH		3.94	5.66	0.58	1.09	943.82

34	EYOPOY		5.04	5.88	0.51	1.13	956.88
35	EYOPUE		5.06	5.97	0.46	1.20	615.64
36	EYOQEP		3.89	6.00	0.48	1.15	583.66
37	EZILUV		3.50	9.32	0.59	0.94	742.01
38	EZIMAC		3.51	9.35	0.59	0.94	732.60
39	FANWIC		7.38	8.81	0.52	1.01	958.31
40	FANWOI		7.76	9.06	0.52	1.00	958.72
41	FAYPUS	DMOF-5	4.37	13.71	0.59	0.84	1351.07
42	FAYRAA	MOF-01	5.87	11.56	0.67	0.72	2205.17
43	FEBQIO	CPF-5	6.26	9.07	0.65	0.89	1975.46
44	FEHCOM		6.03	6.89	0.55	1.04	1092.45
45	FEVFUJ		9.32	10.29	0.61	0.81	1641.61
46	GALBUS		5.58	8.84	0.54	0.96	1203.10
47	GALHUY		4.12	9.51	0.63	0.85	1795.03
48	GIQHAQ		14.16	14.36	0.60	1.19	809.34
49	GITVIP01		5.17	16.70	0.60	1.18	870.01
50	GIVDUL		3.92	5.82	0.53	1.82	528.23
51	GIWNUV		1.80	4.15	0.22	2.21	3.93
52	GUPCOK		3.89	6.63	0.52	1.10	695.37
53	GUPFAZ		4.35	6.46	0.34	1.25	101.09
54	GUSPUG	MFU-5	11.14	12.48	0.47	1.72	402.30
55	HAJKIO		3.35	6.16	0.47	1.36	46.97
56	HASSUR		4.81	6.92	0.60	1.00	1182.91
57	HECQUB		4.52	7.89	0.65	0.79	2499.02
58	HIFVUO		5.40	6.75	0.65	0.82	2134.86
59	HOHMIB		11.22	12.60	0.74	0.61	3061.41
60	IDIWOH		7.01	7.50	0.61	1.00	1517.85
61	IMIXEI		5.10	6.61	0.67	0.81	2342.85
62	ISOJOQ		3.52	5.53	0.53	1.04	581.92
63	JENKIX		6.64	9.18	0.50	1.01	972.52
64	JOKYAJ		3.66	4.75	0.49	1.55	98.93
65	JOKYEN		3.67	4.89	0.49	1.55	296.16
66	JOKYIR		3.63	4.84	0.49	1.56	272.70
67	JOKYOX		3.37	4.76	0.49	1.57	63.82
68	KARLAS	MCF-19-Ia	3.75	7.74	0.56	1.06	639.22
69	KEXFAU		3.31	4.00	0.49	0.91	865.03
70	KEXFIC		3.53	4.71	0.48	1.59	216.02
71	KEYFIF		4.24	4.90	0.32	1.63	107.30
72	KIPKIF		9.79	10.03	0.47	1.11	539.89
73	KOJCUI		4.80	5.32	0.37	1.52	194.88
74	LAJKUE		6.49	15.15	0.51	1.19	741.23
75	LAJLAL		6.52	16.18	0.51	0.99	959.01
76	LAJLEP		6.78	16.17	0.52	1.07	831.99
77	LAJLIT		5.74	14.71	0.51	1.03	1036.63
78	LAMGUB		3.67	4.76	0.48	1.57	54.84
79	LARVIL		6.91	7.14	0.48	2.22	292.34
80	LETFEW		3.69	4.92	0.38	2.53	202.56
81	LUMDIG		4.88	7.13	0.50	1.30	754.15
82	MABJOP		2.69	3.80	0.30	1.57	60.99
83	MABJUV01		2.92	4.01	0.32	1.54	92.28
84	MAFDII		4.38	5.69	0.56	1.30	656.38

85	MIBQAR	MOF-5	7.51	15.02	0.78	0.59	3413.43
86	NAZBAT		3.97	6.62	0.57	1.14	753.69
87	NEFTOJ		3.22	4.42	0.57	1.10	136.05
88	NEVPUA		4.56	6.08	0.39	1.90	286.69
89	NEVQAH		4.50	6.00	0.39	2.06	259.40
90	NEXXIZ		8.90	9.52	0.50	0.89	791.01
91	NEYYOG		10.34	11.67	0.43	2.80	190.46
92	NUJCIE		3.76	4.87	0.48	1.58	86.63
93	OHUKIM		7.88	14.19	0.78	0.42	4339.61
94	OMORUE		4.99	6.24	0.63	0.93	1678.68
95	OTARIL		3.37	5.28	0.41	1.17	191.48
96	OWITAQ		4.86	6.88	0.75	0.66	4888.74
97	OWITEU		4.84	6.84	0.76	0.64	5024.55
98	OWITIY		5.34	7.44	0.77	0.63	5052.08
99	OWITOE		4.79	6.77	0.75	0.65	4960.97
100	OWITUK		4.81	6.80	0.76	0.64	4984.88
101	OWIVAS		4.92	6.85	0.75	0.66	4928.71
102	OWIVEW		4.25	6.79	0.72	0.83	2161.32
103	OYODAI		1.95	9.00	0.51	0.95	1207.69
104	OYODEM		4.35	8.20	0.47	1.04	743.84
105	PAVLUU		4.09	4.52	0.50	2.04	124.58
106	TIRQOB	PCN-13	4.30	5.18	0.47	1.24	753.69
107	KUGZIW	PCN-19	6.69	9.61	0.56	1.00	1335.58
108	DOGBEI	PCN-224-Ni	13.64	23.98	0.81	0.50	3419.42
109	GEGDED	PCN-26	5.02	8.37	0.66	0.90	2043.65
110	NIMPEG	PCN-39	4.53	7.55	0.67	0.65	3227.67
111	PEJNOJ		5.27	5.90	0.68	0.88	2252.98
112	PEVQEO	IRMOF-3-AM3XL	7.38	14.65	0.78	0.61	3385.81
113	RAYKEJ		6.67	9.37	0.49	1.04	867.65
114	RAYKIN		6.38	9.24	0.49	1.07	829.63
115	RAYKUZ		5.69	8.84	0.49	1.10	774.44
116	RAYLAG		5.06	7.28	0.48	1.10	743.15
117	RAYLEK		5.10	7.22	0.48	1.10	783.47
118	RAYLIO		4.94	7.47	0.47	1.12	816.69
119	RAYLOU		5.01	6.68	0.47	1.12	790.18
120	RAYLUA		4.63	6.45	0.47	1.13	668.69
121	RAYMAH		4.60	6.50	0.47	1.13	672.43
122	REGXOS		6.50	18.84	0.64	0.97	1363.18
123	REPCIZ		7.45	8.65	0.65	1.03	1574.74
124	ROGMEG		9.70	12.01	0.75	0.54	3600.38
125	RUHCED		5.80	6.09	0.54	1.95	670.24
126	SAHYIK	MOF-5	7.35	14.81	0.78	0.61	3333.09
127	SAHYOQ		7.49	14.99	0.78	0.59	3429.68
128	SARBOE		3.59	4.78	0.47	1.20	196.13
129	SEGBIR		8.95	9.70	0.46	1.27	536.58
130	SEGBOX		8.91	9.65	0.47	1.38	492.32
131	SUDBOI		3.60	6.29	0.56	1.23	599.80
132	SUGWEX03		3.53	5.77	0.48	1.31	333.84
133	SUTBIT		7.73	8.22	0.62	0.93	1789.41
134	TEQTAM		5.30	6.62	0.47	1.26	781.09

135	TIGDOD		4.20	5.60	0.54	1.03	1290.21
136	TUDJOS	ZIF-8	3.45	10.82	0.56	1.17	829.40
137	TUDMAH		3.75	5.88	0.56	0.81	1124.94
138	TUSGUJ		4.21	10.66	0.61	0.99	1580.88
139	UFANEV		5.22	5.97	0.49	1.40	813.66
140	UHAXUW		3.10	4.53	0.39	1.52	53.64
141	UMUXAC		3.42	5.59	0.36	2.89	42.53
142	UNIGEE		7.33	14.77	0.78	0.61	3362.25
143	UWAGAB03		8.00	11.06	0.72	0.58	3131.08
144	UXEHIP		4.11	5.48	0.52	0.94	917.59
145	UYAQAN		4.76	6.35	0.38	1.14	377.39
146	UYAQER		5.13	6.40	0.38	1.26	510.03
147	VAGMIB		9.52	10.41	0.78	0.50	4227.60
148	VAGMOH		6.93	8.94	0.76	0.50	4542.64
149	VEHKEA		8.64	9.93	0.69	0.88	1750.20
150	VEVJUD		8.45	11.26	0.74	0.94	2020.26
151	VURMOL		5.71	13.62	0.74	0.69	3002.64
152	WEMGAY		4.10	5.32	0.52	1.17	762.00
153	WEMXIX		4.92	5.45	0.36	1.51	106.94
154	XALROT		4.08	7.13	0.62	0.97	1317.36
155	XENZUN		3.62	9.16	0.57	1.08	576.74
156	XIJNUA		15.47	16.04	0.83	0.79	1837.90
157	XIRWEB		3.53	6.49	0.38	1.43	236.84
158	XUNJEW		3.62	7.48	0.47	1.35	526.03
159	YARYEV		3.37	5.09	0.64	1.12	467.64
160	YIZWIN		3.42	4.44	0.48	1.59	238.03
161	YIKJOT		-	-	0.55	1.25	-
162	YIWLOF		5.11	6.91	0.68	2.47	868.62
163	YOZBUL01		10.11	11.96	0.61	1.11	980.87
164	YUVSUE	BIOMOF-11	4.14	4.99	0.48	1.23	533.96
165	VEJZIU	ZIF-10	6.85	12.66	0.67	0.75	2196.35
166	VEJYIT	ZIF-2	4.89	5.84	0.59	0.93	1451.90
167	VEJYOZ	ZIF-3	5.49	7.76	0.62	0.88	1437.80
168	EQOCOC01	ZIF-6	4.83	8.86	0.66	0.76	2630.23
169	GITSUY	ZIF-60	6.84	11.81	0.65	0.77	2047.11
170	GITTUZ	ZIF-68	7.21	9.97	0.56	1.03	781.81
171	GITVAH	ZIF-69	4.78	8.89	0.53	1.15	787.02
172	YOZBOF01	ZIF-79	4.16	7.63	0.51	1.08	778.23
173	YOZCAS	ZIF-81	4.32	8.50	0.53	1.29	631.06
174	WAFKAQ	ZnBDC	5.07	7.28	0.60	0.89	1747.14
175	ZUQPOQ		3.41	4.02	0.33	1.53	122.49

Table S2. Potential parameters of C_2H_6, C_2H_4, and CH_4 molecules used in the simulations

Molecule	ε/k_b (K)	σ (Å)	Bond length (Å)
$\mathbf{C_2H_6}$[1] (CH_3-CH_3)	108.0	3.76	1.54
$\mathbf{C_2H_4}$[1] (CH_2=CH_2)	92.8	3.68	1.33
$\mathbf{CH_4}$[2]	148	3.73	-

1 Y. Wu, H. Y. Chen, D. F. Liu, Y. Qian and H. X. Xi, *Chem. Eng. Sci.*, 2015, **124**, 144−153.

2 M. G. Martin and J. I. Siepmann, *J. Phys. Chem. B*, 1998, **102**, 2569−2577.
